# Acute Effects of a Perturbation-Based Balance Training on Cognitive Performance in Healthy Older Adults: A Pilot Study

**DOI:** 10.3389/fspor.2021.688519

**Published:** 2021-08-16

**Authors:** Dario Martelli, Jiyeon Kang, Federica Aprigliano, Ursula M. Staudinger, Sunil K. Agrawal

**Affiliations:** ^1^Department of Mechanical Engineering, University of Alabama, Tuscaloosa, AL, United States; ^2^Department of Mechanical and Aerospace Engineering, University at Buffalo, New York, NY, United States; ^3^The BioRobotics Institute, Scuola Superiore Sant'Anna, Pisa, Italy; ^4^The Robert N. Butler Columbia Aging Center, Columbia University, New York, NY, United States; ^5^Department of Sociomedical Sciences, Mailman School of Public Health, Columbia University, New York, NY, United States; ^6^Department of Mechanical Engineering, Columbia University, New York, NY, United States; ^7^Department of Rehabilitation and Regenerative Medicine, College of Physicians and Surgeons, Columbia University, New York, NY, United States

**Keywords:** gait, perturbations, balance, cognition, adaptation, aging

## Abstract

Aging is accompanied by an alteration in the capacity to ambulate, react to external balance perturbations, and resolve cognitive tasks. Perturbation-based balance training has been used to induce adaptations of gait stability and reduce fall risk. The compensatory reactions generated in response to external perturbations depend on the activation of specific neural structures. This suggests that training balance recovery reactions should show acute cognitive training effects. This study aims to investigate whether exposure to repeated balance perturbations while walking can produce acute aftereffects that improve proactive and reactive strategies to control gait stability and cognitive performance in healthy older adults. It is expected that an adaptation of the recovery reactions would be associated with increased selective attention and information processing speed. Twenty-eight healthy older adults were assigned to either an Experimental (EG) or a Control Group (CG). The protocol was divided in 2 days. During the first visit, all participants completed the Symbol Digit Modalities Test (SDMT) and the Trail Making Test (TMT). During the second visit, a cable-driven robot was used to apply waist-pull perturbations while walking on a treadmill. The EG was trained with multidirectional perturbations of increasing intensity. The CG walked for a comparable amount of time with cables on, but without experiencing perturbations. Before and after the training, all participants were exposed to diagonal waist-pull perturbations. Changes in gait stability were evaluated by comparing the distance between the heel of the leading leg and the extrapolated Center of Mass (Heel-XCoM Distance—HXD) at perturbation onset (PON) and first compensatory heel strike (CHS). Finally, the cables were removed, and participants completed the SDMT and the TMT again. Results showed that only the EG adapted the gait stability (*p* < 0.001) in reaction to diagonal perturbations and showed improved performance in the SDMT (*p* < 0.001). This study provides the first evidence that a single session of perturbation-based balance training produce acute aftereffects in terms of increased cognitive performance and gait stability in healthy older adults. Future studies will include measures of functional activation of the cerebral cortex and examine whether a multi-session training will demonstrate chronic effects.

## Introduction

Gait, balance, and cognitive disorders are serious problems in late life (Holtzer et al., 2007; Snijders et al., 2007). Aging is generally accompanied by a declining capacity to resolve cognitive tasks (Holtzer et al., 2007; Staudinger, 2015), ambulate (Snijders et al., 2007; James et al., 2016), and react to external balance perturbations (Maki and Mcilroy, 2006; Martelli et al., 2017a). These are regarded as apparent signs of many pathologies leading to falls, a major public health concern for our society (Berg et al., 1997).

The ability to walk and the efficacy of compensatory responses to maintain balance rely not only on the sensorimotor system, but also critically depend on cognitive functioning (Horak, 2006; Snijders et al., 2007; Sturnieks et al., 2012; Morris et al., 2016). Cognitive performance is strongly associated with characteristics of gait and balance (Morris et al., 2016), compensatory responses (Sturnieks et al., 2012), and locomotor adaptability (Caetano et al., 2017). When older adults walk or are exposed to balance perturbations while simultaneously engaged in a cognitively demanding task, performance is impaired in one or both tasks (Woollacott and Shumway-Cook, 2002). The cerebral cortex is directly involved in controlling rapid balance reactions but also keeping the central nervous system prepared to optimize balance recovery reactions even when a future threat to stability is unexpected (Bolton, 2015).

The aptitude to adapt to the environment is essential for walking and compensating for instabilities (Snijders et al., 2007; Caetano et al., 2017). Cognitive (Staudinger, 2015) and locomotor (Bohm et al., 2015; Krishnan et al., 2018) adaptability is still preserved in older age. The control of the compensatory responses required after a balance perturbation can be strengthened (Pai and Bhatt, 2007; Pai et al., 2014; Bohm et al., 2015; Liu et al., 2017). The exposure to repeated external disturbances induces motor adaptations that lead participants to better correct their balance during the recovery phase (i.e., adaptation of the reactive strategy) and modify the volitional control of stability in the face of a possible perturbation (i.e., adaptation of the proactive strategy) (Bohm et al., 2015). As a result, after repeated perturbation-based balance training (PBT) sessions, participants may also show longer-term effects of improved recovery after unexpected loss of balance encountered in daily life, thus reducing their risk for falling (Grabiner et al., 2014; Pai et al., 2014; Mansfield et al., 2015; Gerards et al., 2017; Mccrum et al., 2017; Okubo et al., 2017). Similarly, single bouts of moderate exercise are able to induce acute physiological responses that have a positive impact on the brain and on cognitive performance, as assessed by behavioral measures (Lambourne and Tomporowski, 2010; Chang et al., 2012; Netz, 2019). Cumulative effects of exercise have been associated with increases in brain volume (Colcombe et al., 2006; Hillman et al., 2008; Herold et al., 2019) and cognitive performance (Angevaren et al., 2008; Smith et al., 2010), more efficient brain functioning (Voelcker-Rehage et al., 2011), and attenuated cognitive decline (Lautenschlager et al., 2012).

To our knowledge, the impact of exposure to repeated balance perturbations on cognitive performance has not yet been studied. The compensatory reactions generated to control dynamic balance in response to external perturbations are not merely segmental reflexes organized at the level of the spinal cord, but rather depend on the integration of proprioceptive, visual, and vestibular information implicating many levels of the central nervous system (Horak, 2006; Maki and Mcilroy, 2006; Jacobs and Horak, 2007; Bolton, 2015; Varghese et al., 2017). Cognitive resources are needed to recognize a disturbance of balance and then rapidly initiate a recovery step, maintain balance on a single limb, and navigate the contralateral limb to regain stability. Besides the sense of balance, it requires specific cognitive abilities such as selective attention and speed of information processing (Snijders et al., 2007). This suggests that training dynamic balance recovery reactions also should show acute cognitive training effects.

The present study aims to investigate to what extent the exposure to repeated balance perturbations while walking can produce acute improvements in gait stability and cognitive performance in community-dwelling, healthy older adults. We hypothesized that a single-session of gait training encompassing unpredictable waist-pulls would be more effective than unperturbed walking in improving reactive and proactive control of gait stability and cognitive performance in terms of information processing speed and selective attention.

## Materials and Methods

### Participants

Twenty-eight healthy community-living older adults were randomly assigned to either the Experimental Group (EG: 14 subjects, 3 males) or the Control Group (CG: 14 subjects, 3 males). Inclusion criteria included: (i) living independently in the community, (ii) at least 65 years old; (iii) absence of acute, severe, or unstable medical illness; (iv) not reporting any significant neural, muscular, or skeletal disease, and (v) able to safely walk on a treadmill without mobility aids. Participants were informed about the research procedure and signed a written consent form approved by the Institutional Review Board of Columbia University, before participating.

### Procedure

**Figure 1:**
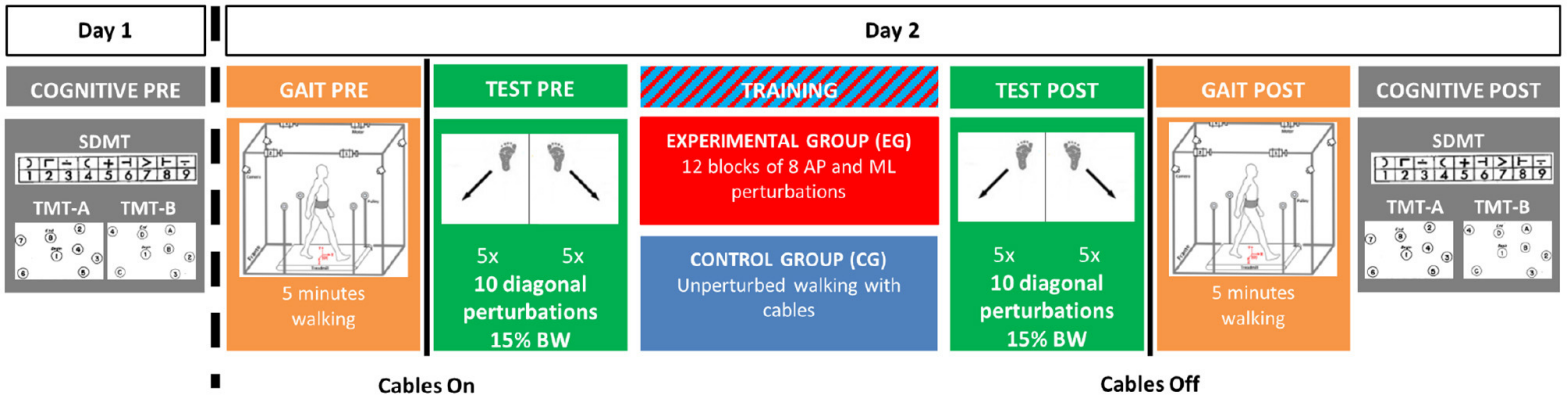
Experimental Protocol. The experiment was broken in two visits. During Day 1, (COGNITIVE PRE), participants completed the Symbol Digit Modalities Test (SDMT) and the Trail Making Test Part A (TMT-A) and Part B (TMT-B). During Day 2, participants first walked without cables at their preferred speed on the treadmill for 5 minutes (GAIT PRE). Then, cables were attached, and they were exposed to 10 diagonal perturbations (TEST PRE). In the TRAINING session, the Experimental Group (EG) was trained with 12 blocks of 8 antero-posterior (AP) and medio-lateral (ML) perturbations of increasing intensity. The Control Group (CG) walked on the treadmill with the cables attached for the same amount of time as the EG (i.e., 24 minutes). Both groups were then exposed to the same set of perturbations delivered before the training session (TEST POST). Then, the cables were removed, and all participants walked again for 5 minutes (GAIT POST). Finally, after a 10-minute resting period, all participants completed the same cognitive tests of Day 1 (COGNITIVE POST).

**Figure 2:**
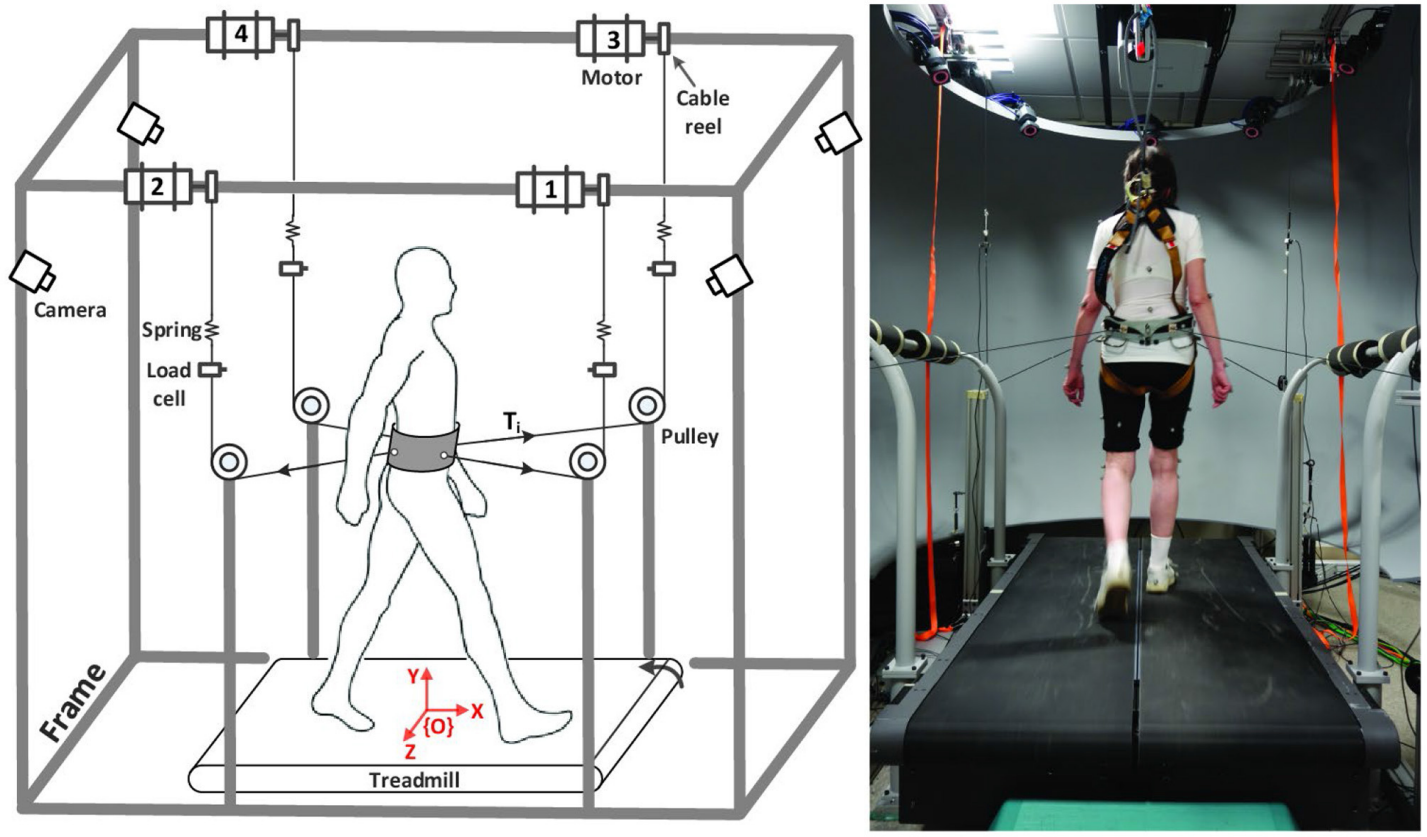
Experimental Setup. Schematic of the Active Tethered Assistive Pelvic Device (A-TPAD) and a picture of a participant while walking with it. Four AC servo motors are mounted on a rigid frame and connected through cables to a fabric hip belt worn by the subject. A load cell and a spring are installed in series with each cable. A closed-loop controller ensures delivery of the correct tensions in the motors. Cables are routed using pulleys to be diagonally directed. The heights of the pulleys were changed for each subject such that during standing each cable was almost parallel to the floor (range: 5°, −20°). Participants walk at constant speed on a split-belt treadmill (Bertec Instrumented Treadmill). A ten-camera motion capture system (Vicon Bonita-10 series), the load cells, and the force plates embedded in the treadmill are used as a part of the controller. The motion capture system is used to track the cable orientation. The force plates are used to detect heel strikes in real time (vertical ground reaction force threshold at 50N) and time the application of perturbations. When cables were attached to the subject, a constant force of 25N is applied by each motor to prevent cable slackening. Waist-pull perturbations with peak force of a desired amplitude proportional to the subject's body weight (BW) are provided by applying a transient pulse on one or two of the four cables. The controller is implemented on a LabVIEW, (National Instrument, PXI real time system).

Participants were asked to come to the lab on two occasions (Figure 1). During the first session, they completed questionnaires describing their study cohort, a battery of functional tests and they were tested for baseline cognitive performance (COGNITIVE PRE) using the Symbol Digit Modalities Test (SDMT) (Sheridan et al., 2006) and the Trail Making Test (TMT) (Tombaugh, 2004). During the second session, usually occurring within 1 week of the first, the experimental intervention took place using the Active Tethered Pelvic Assist Device (A-TPAD), an innovative cable-driven robot conceived for gait rehabilitation able to apply controlled force-moments at the human pelvis in any direction and precise instants of the gait cycle (Vashista et al., 2015) (Figure 2). In this configuration, the A-TPAD is used to apply multidirectional waist-pull perturbations while walking on a treadmill (Martelli et al., 2016, 2017b,c, 2018).

Participants were equipped with the pelvic brace necessary to apply the perturbations, a harness to protect them from falling, and reflective markers that allow to collect kinematic data (Figure 2). Preferred treadmill walking speed was determined for each participant and then maintained during the experiment. Speed was determined by gradually increasing the speed by 0.1 m/s until the subject reported that was too fast and then reducing it by 0.1 m/s. All participants first walked on the treadmill for 5 min. Subsequently, cables were attached to the brace. All subjects were exposed to 10 diagonal perturbations while walking (TEST PRE). Perturbations consisted of 5 pulls with Motor 2 (back-right perturbation) triggered at right heel strike and 5 pulls with Motor 4 (back-left perturbation) triggered at left heel strike (Figure 2, left panel). The first perturbation was delivered at right heel strike and then the order of perturbations was alternated. Peak force was fixed at 15% of the Body Weight (BW). Then, the EG was exposed to 12 blocks of 8 Antero-Posterior (AP) and Medio-Lateral (ML) perturbations of increasing intensities (TRAINING). In each block, 4 directions (forward, backward, leftward and rightward), and 2 events (right and left heel strikes) were used. At the beginning, the peak force was 15% and 5% BW for AP and ML perturbations, respectively. Every four blocks, the peak force was increased by 5% BW. The order of the perturbations in each block was chosen randomly. The range of intensity of the perturbations was determined based on previous experiments with healthy young subjects (Martelli et al., 2016, 2017b, 2018). The CG did not receive any perturbation during the training session, but they walked on the treadmill with the cables attached for the same amount of time as the EG. In order to reduce the risk of fatigue, the treadmill was stopped every four blocks (for the EG) or 8 min (for the CG) and subjects were told they could rest at any time if they felt tired. All participants were then exposed to the same set of perturbations delivered before the training session (TEST POST). All perturbations were delivered while walking at constant speed and consisted of a trapezoidal force profile (rise, hold and fall times of 150 ms duration each). The time between perturbations was chosen randomly (5–15 s). Participants were aware that they could be perturbed at the waist when the cables were attached, but were not informed about the magnitude, the direction or the timing of the perturbations. Before the intervention started, they were instructed to maintain balance and keep walking. Then, the cables were removed, and all participants walked for another 5 min (GAIT POST). For all the duration of the experiment, subjects wore a safety harness to prevent them from falling but without restricting their movements. Finally, after a 10-min resting period, all participants completed the SDMT and the TMT as in the first session (COGNITIVE POST). All participants completed the experiment without difficulty. Inspection of video recording images confirmed that all participants were able to recover their balance without being assisted by the safety harness. Technical problems resulted in missing the TEST POST data for one participant in the CG group.

### Measures

#### Cognitive Performance

The SDMT requires individuals to identify nine different symbols corresponding to the numbers 1 through 9, and manually fill the blank space under each symbol with the corresponding number as fast as possible. Two scores were calculated: total number of correct answers given in 90 s (SDMT-C), and time to complete all 110 blank spaces (SDMT-T). The TMT consists of two parts (TMT-A and TMT-B). TMT-A requires an individual to draw lines sequentially connecting 25 encircled numbers distributed on a sheet of paper. Task requirements are similar for TMT-B except the person must alternate between numbers and letters (e.g., 1, A, 2, B, 3, C, etc.). The score on each part represents the amount of time required to complete the task without considering the number of mistakes. In case an error was made, the participant was instructed to return to the “circle” where the error originated and continue. Participants were instructed to complete each test as quickly and accurately as possible.

#### Biomechanical Measures

The trajectories of 55 reflective markers were collected at 200 Hz using a 10-camera motion capture system (Vicon Bonita-10 series). Missing kinematic data were estimated by means of cubic spline interpolations. High-frequency related noise was removed from digitized coordinates by low-pass filtering data (zero-lag, fourth-order Butterworth low-pass filter) with a cut-off at 10 Hz. Heel strikes were determined as the first point in the descending phase of the lateral malleolus' marker in which the vertical position did not decrease more than 2 mm for two consecutive time frames (Alton et al., 1998). Missing or false gait events were manually checked. A 13-segment biomechanical model was used to calculate the trajectory of the body Center of Mass (CoM) (Martelli et al., 2017c).

In order to maintain balance, it is necessary to control the relative position and velocity between the moving body's CoM and the moving base of support (BoS) (Patla, 2003; Hof, 2008). The extrapolated center of mass (XCoM) (Hof et al., 2005; Hof, 2008) represents the state of the CoM when taking into account both its position and velocity and was calculated as:

$$\begin{array}{l} {\text{XCoM}}_{\text{x,y}}={\text{CoM}}_{\text{x,y}}+{\text{VCoM}}_{\text{x,y}}/\sqrt{g/l} \end{array}$$

where CoM_x,y_ and VCoM_x,y_ are the AP and ML components of the CoM position and velocity vectors, *l* is the estimated pendulum length based on the instantaneous distance between the body CoM and the ankle joint of the leading leg and *g* is the gravitational acceleration. The VCoM_x,y_ was calculated as the first derivative of CoM_x,y_ by using the three-point central differences method. The treadmill speed was added to the VCoM_x_.

Gait stability was quantified during the TEST PRE and TEST POST using the 2D Euclidean distance between the heel marker of the leading leg and the XCoM (i.e., Heel-XCoM Distance—HXD). Two events were identified: Perturbation onset (PON) and the compensatory heel strike (CHS—first heel strike after PON). HXD-PON was used to identify stability before the perturbation started and possible proactive adaptations in the gait pattern (note that at PON the perturbation force is still at zero). HXD-CHS was used to identify stability after the perturbation and possible reactive adaptations of the early compensatory reaction.

#### Anthropometric, Socio-Demographic, and Functional Measures

Further assessments were performed during the first visit to verify that participants' age, body height, body weight, socio-demographic, and levels of balance, mobility, and fear of falling were comparable. Socio-demographic data were assessed with a self-report questionnaire that provided information regarding education, family status, housing, occupation, life satisfaction and general health status. The life satisfaction and general health subparts score ranged from 1 (low satisfaction/health) to 5 (high satisfaction/health). Functional measures included the Berg Balance Scale (BBS) (Berg et al., 1992), the Short Physical Performance Battery (SPPB) (Guralnik et al., 1994), and the Falls Efficacy Scale International (FES-I) (Yardley et al., 2005).

### Statistical Analysis

Anthropometric characteristics (i.e., age, body height, body mass), preferred treadmill speed, levels of health status and life satisfaction, scores obtained in the BBS, SPPB and FES-I in the two groups were compared by means of independent samples *t*-tests. Level of education was compared by the Kruskal-Wallis test.

Mixed design analyses of variance (ANOVAs) were performed using the HXD and cognitive test scores (SDMT-C, SDMT-T, TMT-A, and TMT-B) as dependent variables. For the cognitive test scores, a 2-way ANOVA was used. Group (EG and CG) and session (PRE and POST) were used as between- and within-subject factors, respectively. For the HXD, a preliminary ANOVA was performed to confirm that values were similar for perturbations delivered at right or left heel strikes. Since that no significant effect of side was detected, the average value obtained during the 5 perturbations delivered at right heel strike was used in the analysis. A 3-way ANOVA was used with an additional within-subject factor: the time of the gait cycle (PON and CHS) at which the HXD was evaluated. At TEST PRE, it is expected that all participants would show a significant difference between HXD-PON and HXD-CHS due to the effect of the waist-pulls. At TEST POST, it is expected that the CG would still show differences while the EG—that has been exposed to repeated multidirectional perturbations during the TRAINING—would be able to adapt gait stability and “cancel” the effect of the perturbation in a single step, such that the HXD at CHS would be similar to the HXD at PON. Given the exploratory nature of the study, the alpha level of the ANOVAs tests was not adjusted. Significant interaction effects were followed up by Tukey's Honest significance tests. The Lilliefors test, Levene's test for equality of error variances, and the Mauchly's tests were performed to check the normality, homoscedasticity, and sphericity assumptions, respectively. Statistical significance was set at *p* < 0.050.

## Results

Table 1 describes characteristics EG and CG. The two groups did not differ on any of the sample characteristics (*p* > 0.539, Table 1). All participants reported high levels of subjective well-being and subjective health and were positively biased toward higher levels of education.

**Table 1:** Descriptive statistics^a^ of participants in Experimental Group (EG) and Control Group (CG).

	**Experimental Group (EG)** **(*n* = 14)**	**Control Group (CG)** **(*n* = 14)**	***p*-values**
Age [years]	70.7 ± 3.7	71.6 ± 4.7	0.598
Height [m]	1.66 ± 0.73	1.65 ± 0.80	0.652
Body mass [kg]	70.5 ± 14.4	71.6 ± 12.6	0.824
Treadmill Speed [m/s]	0.83 ± 0.18	0.83 ± 0.20	1.000
BBS [0–56]	55.0 ± 1.8	54.9 ± 1.1	0.903
SPPB [1–12]	10.7 ± 1.2	10.4 ± 1.2	0.539
FES-I [16–64]	21.1 ± 5.3	21.4 ± 4.7	0.881
Life Satisfaction [1–5]	4.3 ± 0.7	4.4 ± 0.6	0.633
Subjective Health [1–5]	4.5 ± 0.5	4.2 ± 0.6	0.194
Education [≤Highschool, >Highschool]	7.1, 92.9%	7.1, 92.9%	1.000

a *Values are reported as Means ± Standard Deviations. BBS, Berg Balance Scale; SPPB, Short Physical Performance Battery; FES-I, Falls Efficacy Scale International*.

**Figure 3:**
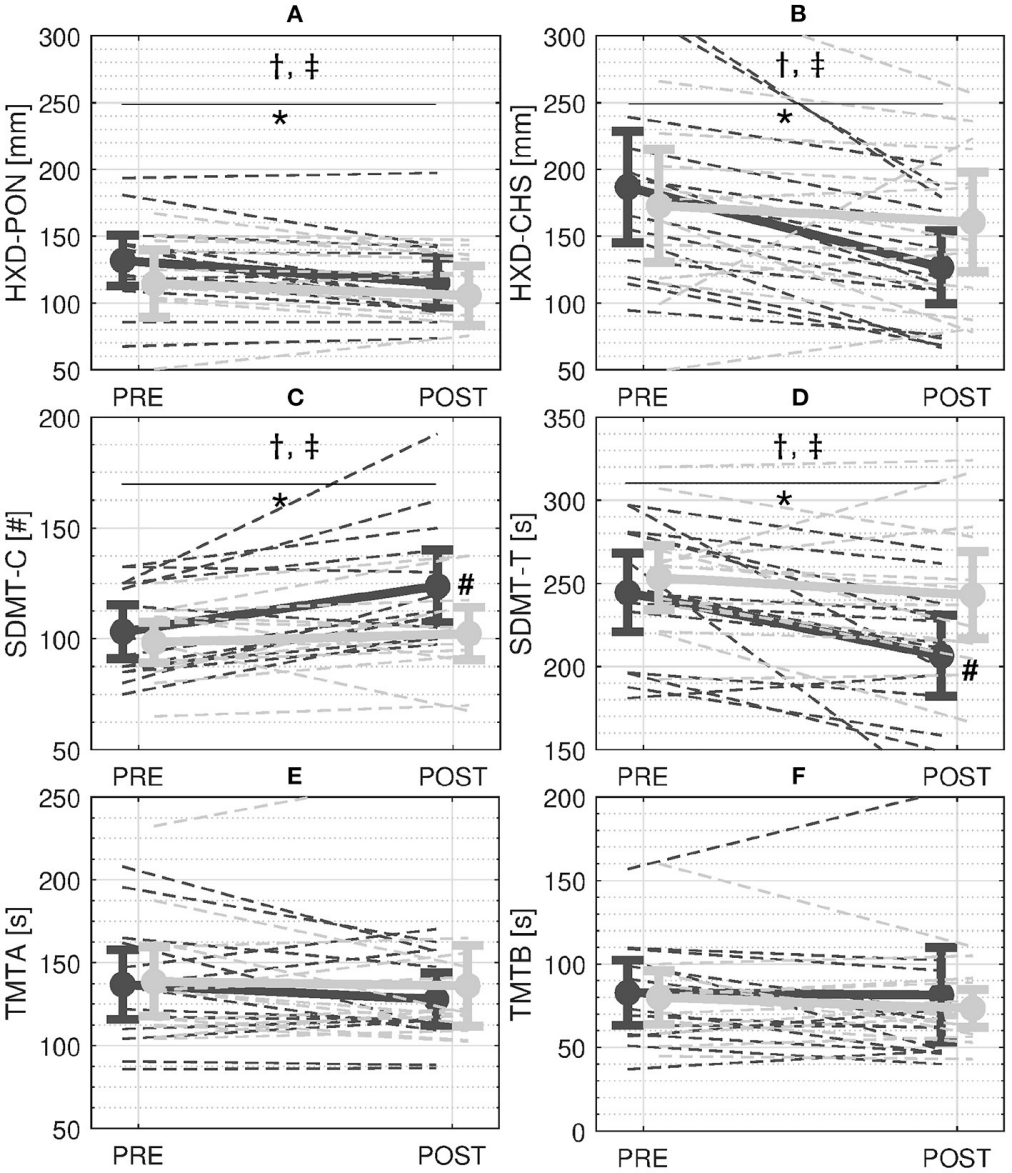
Results. **A** and **B**: Stability measures during perturbed walking with cables—Average Heel-XCoM Distance (HXD) at perturbation onset (PON) **(A)** and compensatory heel strike (CHS) **(B)**. **C** and **D**: Scores of the Symbol Digit Modalities Test (SDMT)—Average number of correct answers in 90 s (SMDT-C) **(C)** and time to complete 110 answers (SMDT-T) **(D)**. **E** and **F**: Scores of the Trail Making Test (TMT)—Time to complete part A (TMT-A) **(E)** and time to complete part B (TMT-B) **(F)**. Solid and dashed dark and light gray lines represent the mean and single-subject changes in the Experimental Group (EG) and the Control Group (CG) from PRE to POST, respectively. Bars refer to 95% confidence interval of the mean. †, ‡ symbols indicate a significant main effect of session and group×session interaction term respectively. *and # symbols indicate a significant effect of the Tukey's Honest significance test for the within-subject and between-subject factors, respectively.

At PRE, participants showed an average HXD-PON of 123.5 ± 38.7 mm. Waist-pull perturbations caused a disruption of normal walking, such that, at the following heel strike, participants showed an HXD-CHS of 182 ± 7 mm. The results of the ANOVA revealed that the HXD showed a significant effects of session (*p* < 0.001), time of the gait cycle (*p* < 0.001), group×session interaction term (*p* = 0.006), time×session interaction term (*p* = 0.019), and group×session×time interaction term (*p* = 0.030, Figures 3A,B). Further analysis revealed that: (i) the HXD changed from PRE to POST for participants in the EG (PRE: 160.4 ± 61.9 mm; POST: 122.5 ± 39.8 mm; *p* < 0.001) but not for the participants in the CG (PRE: 145.8 ± 67.0 mm; POST: 136.7 ± 56.2 mm; *p* = 0.431); (ii) both HXD-PON (PRE: 132.1 ± 32.9 mm; POST: 115.6 ± 31.5 mm; *p* = 0.005) and HXD-CHS (PRE: 188.8 ± 71.8 mm; POST: 129.4 ± 46.9 mm; *p* < 0.001) showed significant changes from PRE to POST for the EG; (iii) due to the steep decrements of HXD-CHS at POST, significant differences between HXD-PON and HXD-CHS for the EG were observable only at PRE (*p* = 0.010) but not at POST training (*p* = 0.686). On the contrary, neither HXD-PON (PRE: 115.0 ± 43.2 mm; POST: 106.2 ± 35.8 mm; *p* = 0.680) and HXD-CHS (PRE: 176.5 ± 73.7 mm; POST: 167.3 ± 57.3 mm; *p* = 0.951) showed significant changes from PRE to POST for the CG. As a result, the HXD-CHS and the HXD-PON were significantly different at both PRE and POST sessions for the CG (*p* < 0.006).

In regards to the cognitive tests, the ANOVA revealed significant effects of the session (SDMT-C: *p* = 0.001; SDMT-T: *p* < 0.001) and group×session interaction term (SDMT-C: *p* = 0.022; SDMT-T: *p* = 0.040) for both SDMT-C and SDMT-T. Further analysis showed that: (i) only the EG increased the number of correct answers given in 90 s from PRE to POST (PRE: 41.3 ± 8.4; POST: 49.5 ± 11.3; higher SDMT-C, *p* < 0.001, Figure 3C); (ii) only the EG completed the 110 items more quickly from PRE to POST (PRE: 244.5 ± 41.1 sec; POST: 206.5 ± 42.1 sec; lower SDMT-T, *p* < 0.001, Figure 3D); and (iii) the EG showed a higher SDMT-C (*p* = 0.030, Figure 3C) and lower SDMT-T (*p* = 0.036, Figure 3D) compared to the CG at POST. On the contrary, the CG did not modify neither SDMT-C (PRE: 39.4 ± 6.3; POST: 40.9 ± 8.2, *p* = 0.421) or SDMT-T from PRE to POST (PRE: 253.4 ± 33.3 sec; POST: 243.1 ± 45.3 sec, *p* = 0.264, Figure 3D). No significant main or interaction effects of group and session were found for either the TMT-A (*p* > 0.203, Figure 3E) or the TMT-B (*p* > 0.086, Figure 3F).

## Discussion

This study aimed to investigate if the exposure to repeated balance perturbations delivered while walking would induce acute adaptations of gait stability and cognitive performance in community-dwelling, healthy older adults. Research on cognitive and locomotor adaptability during balance-demanding tasks is highly important, as it may contribute to the design of effective methods to early detect and remediate gait and cognitive deficits. As hypothesized, results showed that the exposure to multidirectional waist-pull perturbations induced acute modifications of the (i) recovery reaction in terms of stability both before and after the perturbation onset; and (ii) cognitive task performance, as measured by the SDMT. This study provides the first evidence that systematic perturbations of gait induce acute changes in cognitive functioning.

Replicating our previous results (Martelli et al., 2017b,c, 2018), we showed that participants in the EG were able to adapt their capacity to counteract diagonal waist-pull perturbations. Both reactive (HXD-CHS) and proactive (HXD-PON) adaptations in the EG were primarily accounted for by a reduced distance between the XCoM of the body and the heel of the leading leg (i.e., lower HXD-PON and HXD-CHS at POST, Figures 3A,B). Such changes allowed the EG to compensate for the instability created by the waist-pull in a single step (i.e., at POST, the HXD at CHS was similar to the HXD at PON). The ability to better control the relationship between the XCoM and the BoS while walking and in reaction to different kinds of perturbations has been shown in young adults as compared to older adults (Bierbaum et al., 2010) and has been associated with a reduced risk of falling (Lugade et al., 2011). It can be argued that proactive adjustments were made predominantly with feedforward control implemented by the central nervous system to increase stability before the perturbation actually started (Bhatt et al., 2006). This modification was beneficial to start the reaction to the perturbation from a more stable position and ideally reduce the reliance on the reactive corrections after the onset of the perturbation. Reactive adjustments to external unanticipated perturbations are largely influenced by the central nervous system as well (Horak, 2006; Jacobs and Horak, 2007; Bolton, 2015; Varghese et al., 2017). The recovery reactions against repeated balance perturbations can bypass some stages of information processing due to a change in the central set developed from prior experience (Horak, 2006). These fast responses recalibrate a previously constructed motor memory without the need of developing a new motor pattern. After the initiation of the compensatory reaction, the cerebral cortex can also modulate late-phase or change-in-support responses characteristics through direct control (Bolton, 2015).

As hypothesized, participants in the EG also showed acute changes in cognitive functioning. The compensatory reactions to perturbations delivered while walking are characterized by fast changes in the base of support that requires challenges of spatial navigation, coordination and affordances in the surrounding environment (Maki and Mcilroy, 2007). The amount of sensorimotor and cognitive processing required to maintain balance and the specific domains involved depend on the type and complexity of the task. We can assume that exposure to repeated perturbations may have been linked with increased activation of cognitive control processes, especially the ones dedicated to processing speed, integration of motion, and navigation to a higher degree than unperturbed walking (Snijders et al., 2007; Sturnieks et al., 2012; Senden et al., 2014; Patel and Bhatt, 2015; Wittenberg et al., 2017). This cognitive activation may have continued to facilitate the speed of mental processes in the EG once the cables were removed and may have contributed to the improvements in the SDMT.

For the first time, we were able to show that a single session of perturbation-based balance training (PBT) can affect cognitive performance in older adults. In relation to the exercise-cognitive relationship, activities can be classified into physical (i.e., aerobic and strength) and motor training (i.e., balance, coordination and flexibility) (Netz, 2019). Studies that compared the cognitive improvements of motor and physical training concluded that both are beneficial, but motor training may better stimulate changes in information processing, especially the ability to handle visual and spatial information (Paffenbarger et al., 2001; Netz, 2019). Another important difference between these two training modes is the driving mechanism that affects the cognitive function (Netz, 2019). During physical training, it is the intensity of the exercise that influences neuroplasticity. In contrast, during motor training, it is the complexity of the task that has an effect on cognitive improvements (Carey et al., 2005; Pesce, 2012; Netz, 2019). PBT falls inside the second category of exercise mode and it can be considered as a motor training task with high complexity and neuromuscular demands. Our preliminary results confirm that balance training is effective in improving speed of information processing and the introduction of unanticipated balance perturbations in the task could be particularly beneficial for this domain of cognitive functioning. However, more research is needed to assess the dose-response relationship between level of complexity and cognition for motor activities (Netz, 2019). While intensity is measurable, complexity is hard to measure, and thus the dose-response effect of motor activities on cognition is difficult to determine. PBT is usually implemented with platforms able to impose controlled, standardized and repeatable perturbations (Mccrum et al., 2017). Therefore, they could be ideal for analyzing the dose-response relationship by comparing the effects on cognitive performance of PBT sessions in which complexity is controlled by adjusting the amplitude of balance perturbations. Future studies will further investigate this critical aspect.

Even though in this study no measures of cortical neurophysiological functioning were collected, we can speculate that the repeated exposure to perturbations may have caused changes in brain activity, the so-called perturbation-evoked response (Bolton, 2015; Mierau et al., 2015; Varghese et al., 2017), that were functional to improve the performance in the SDMT. A number of cognitive structures become activated in response to both expected and unexpected perturbations including brain areas generally considered to be involved in executive control such as the pre-frontal cortex and the fronto-central cortical region (Bolton, 2015; Patel et al., 2018). Similarly, the SDMT requires recruiting cerebral networks interconnecting fronto-parietal areas related to selective attention processes, occipital areas related to visual attention, the cerebellum (Forn et al., 2013), and the anterior and posterior corpus callosum known to connect to pre-frontal, parietal and motor cortical areas involved in sensory integration, decision making and motor response (Gawryluk et al., 2014).

Despite an increment in the SDMT, both TMT-A and TMT-B did not show any significant modifications in both groups (Figures 3E,F). This may be because the cognitive mechanisms that underlie the TMT are not the ones that are mainly involved while reacting to balance perturbations. Even if the TMT is one of the most widely used instruments in neuropsychological assessment as an indicator of speed of cognitive processing and executive function, an in depth analysis reveals that the TMT-A requires mainly visuoperceptual abilities, and the TMT-B primarily reflects working memory and secondarily task-switching ability (Sanchez-Cubillo et al., 2009). On the contrary, the SDMT is a neuropsychological test with high reliability and ideal to measure information processing speed and selective attention. This is because it is an easy task involving a short time-frame, and carrying out it does not allow use of alternative strategies as is often the case for other tasks indexing executive functioning (Forn et al., 2013).

Even though the results of this study are promising, several limitations need to be considered when interpreting results. Our sample of 28 older adults was rather small which limited the power of the statistical tests. Yet, the fact that nevertheless significant group differences were found is encouraging. Preferred treadmill speed was slow for both groups and equal to 0.83 m/s. This may be related to the procedure used to determine it. Participants were reminded that the walking speed would stay the same throughout the experiment once determined. This may have led the participants to choose a more conservative speed as ‘too fast' to avoid getting tired. In other words, we think that this is more of a psychological than a functional effect. This assumption is supported by the fact that for both groups the BBS and the SPPB showed performance higher than 54 (out of 56) and 10 (out of 12) points. These scores would rank both groups as highly functioning older adults with low risk of falls (Bogle Thorbahn and Newton, 1996; Veronese et al., 2014). Determining the preferred walking speed on the treadmill was a measure to tailor the experimental procedure to the respective participant. We do not think that walking at slower speed influenced the results concerning the impact of the gait perturbation intervention. Similar experiments, without testing the cognitive function, were conducted with healthy young subjects (Martelli et al., 2016, 2017b). In these experiments, young participants walked at a speed of about 1.1 m/s. Results were similar and showed an adaptation of the post-training gait pattern as well as the recovery reactions. Accordingly, we do not expect that a faster walking speed for older participants would have yielded a different outcome. Moreover, by keeping walking speed slow, we further reduced the risk of fatigue, a possible confounding effect on cognitive and motor performance. Even if the effects of acute physical fatigue on cognitive performance post-exercise have been unclear (Brisswalter et al., 2002; Lambourne and Tomporowski, 2010), several factors associated with peripheral fatigue could lead to the appearance of central fatigue and a decrease in cognitive performance. Further studies should control for metabolic expenditure and analyze the propensity and functional implications of fatiguability.

We only included two cognitive tests, consequently we are not able to delineate more precisely which dimensions of cognitive aging may profit from the gait perturbation intervention and which may not. In addition, we did not include any specific cognitive screening to determine cognitive impairments. However, performance levels in the SDMT and TMT were similar to the ones obtained by non-clinical adults of similar age in the literature (Tombaugh, 2004; Sheridan et al., 2006) (Figures 3C–F). Accordingly, we can infer that our sample was not characterized by any severe cognitive deficits. The same cognitive test was presented at baseline and post-training, thus creating possible practice effects (Goldberg et al., 2015). The introduction of a control group partially obviated this problem, yet we cannot rule out that the perturbation-based balance training facilitated the learning of solving the task rather than processing speed itself. Further studies involving larger samples, a wider range of older adults, multiple baseline assessments and additional cognitive tests are necessary to confirm our conclusions. Moreover, we cannot reject the hypothesis that the perturbations experienced by the EG may have also increased participants' vigilance and arousal level as compared to the CG. Possibly such arousal could have been maintained through the resting period after the intervention and facilitated speeded mental processes and improved cognitive task performance (Lambourne and Tomporowski, 2010). Future studies should therefore include an attention control group to better tease apart arousal from sensorimotor effects on cognitive improvements. Moreover, both the participants and the investigators should be blinded to group assignment to ensure objectivity. Future studies should also include measures of cortical function to investigate directly alterations of the brain function. Finally, the present study focused on acute and not lasting effects. Future studies have to examine whether a multi-session and more extensive training will demonstrate chronic effects on walking balance and cognitive performance.

## Data Availability Statement

The raw data supporting the conclusions of this article will be made available by the authors, without undue reservation.

## Ethics Statement

The studies involving human participants were reviewed and approved by the Institutional Review Board of Columbia University. The participants provided their written informed consent to participate in this study.

## Author Contributions

DM, US, and SA conceptualized and designed the study. DM, JK, and FA collected the data and worked on the hardware and software of the system. DM cleaned and analyzed the data, performed the statistical analysis, prepared a first draft of the manuscript, and created the figures and tables. US and SA supervised the project. All authors contributed extensively to the work presented in this paper, commented on the manuscript throughout the editorial process, and approved the final submitted version.

## Conflict of Interest

The authors declare that the research was conducted in the absence of any commercial or financial relationships that could be construed as a potential conflict of interest.

## Publisher's Note

All claims expressed in this article are solely those of the authors and do not necessarily represent those of their affiliated organizations, or those of the publisher, the editors and the reviewers. Any product that may be evaluated in this article, or claim that may be made by its manufacturer, is not guaranteed or endorsed by the publisher.
